# An active learning based classification strategy for the minority class problem: application to histopathology annotation

**DOI:** 10.1186/1471-2105-12-424

**Published:** 2011-10-28

**Authors:** Scott Doyle, James Monaco, Michael Feldman, John Tomaszewski, Anant Madabhushi

**Affiliations:** 1 Biomedical Engineering Department, Rutgers University, Taylor Road, New Jersey, USA; 2 Department of Surgical Pathology, University of Pennsylvania, Pennsylvania, USA

## Abstract

**Background:**

Supervised classifiers for digital pathology can improve the ability of physicians to detect and diagnose diseases such as cancer. Generating training data for classifiers is problematic, since only domain experts (e.g. pathologists) can correctly label ground truth data. Additionally, digital pathology datasets suffer from the "minority class problem", an issue where the number of exemplars from the non-target class outnumber target class exemplars which can bias the classifier and reduce accuracy. In this paper, we develop a training strategy combining active learning (AL) with class-balancing. AL identifies unlabeled samples that are "informative" (i.e. likely to increase classifier performance) for annotation, avoiding non-informative samples. This yields high accuracy with a smaller training set size compared with random learning (RL). Previous AL methods have not explicitly accounted for the minority class problem in biomedical images. Pre-specifying a target class ratio mitigates the problem of training bias. Finally, we develop a mathematical model to predict the number of annotations (cost) required to achieve balanced training classes. In addition to predicting training cost, the model reveals the theoretical properties of AL in the context of the minority class problem.

**Results:**

Using this class-balanced AL training strategy (CBAL), we build a classifier to distinguish cancer from non-cancer regions on digitized prostate histopathology. Our dataset consists of 12,000 image regions sampled from 100 biopsies (58 prostate cancer patients). We compare CBAL against: (1) unbalanced AL (UBAL), which uses AL but ignores class ratio; (2) class-balanced RL (CBRL), which uses RL with a specific class ratio; and (3) unbalanced RL (UBRL). The CBAL-trained classifier yields 2% greater accuracy and 3% higher area under the receiver operating characteristic curve (AUC) than alternatively-trained classifiers. Our cost model accurately predicts the number of annotations necessary to obtain balanced classes. The accuracy of our prediction is verified by empirically-observed costs. Finally, we find that over-sampling the minority class yields a marginal improvement in classifier accuracy but the improved performance comes at the expense of greater annotation cost.

**Conclusions:**

We have combined AL with class balancing to yield a general training strategy applicable to most supervised classification problems where the dataset is expensive to obtain and which suffers from the minority class problem. An intelligent training strategy is a critical component of supervised classification, but the integration of AL and intelligent choice of class ratios, as well as the application of a general cost model, will help researchers to plan the training process more quickly and effectively.

## Background

### Motivation

In most supervised classification schemes, a training set of exemplars from each class is used to train a classifier to distinguish between the different object classes. The training exemplars (e.g. images, pixels, regions of interest) usually have a semantic label assigned to them by an expert describing a category of interest or class to which they belong. Each training exemplar serves as an observation of the domain space; as the space is sampled more completely, the resulting classifier should achieve greater classifier accuracy when predicting class labels for new, unlabeled (unseen) data. Thus, typically, the larger the training set, the greater the accuracy of the resulting classifier [1]. In most cases, the training set of labeled data for each of the object categories is generated by a human expert who manually annotates a pool of unlabeled samples by assigning a label to each exemplar.

**Figure 1:**
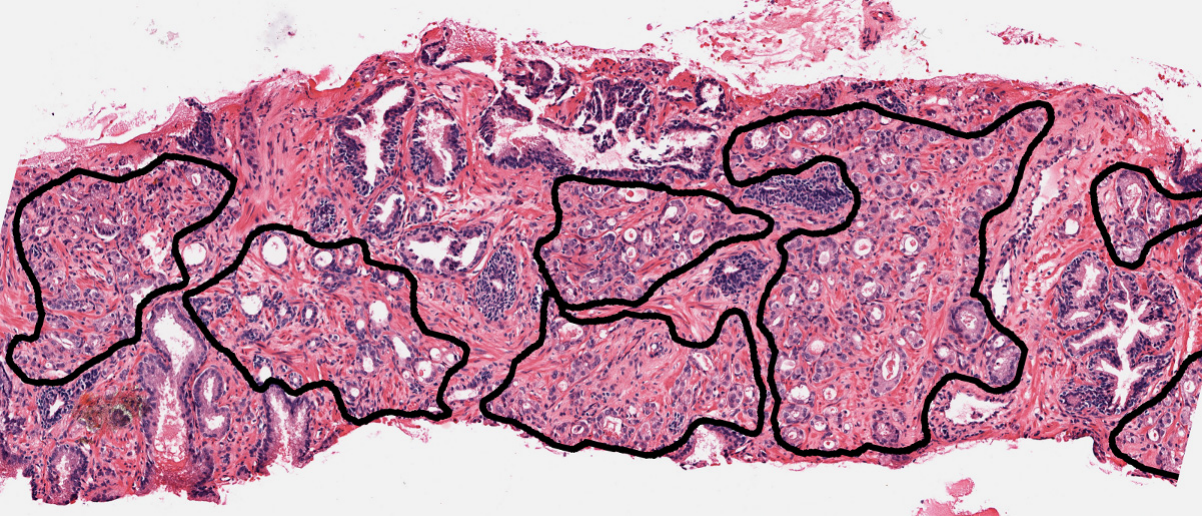
**Annotated Prostate Biopsy Tissue Image**. Annotation of CaP (black contour) on digital histopathology. CaP tissue often appears near and around non-CaP tissue, making annotation difficult and time-consuming.

**Figure 2:**
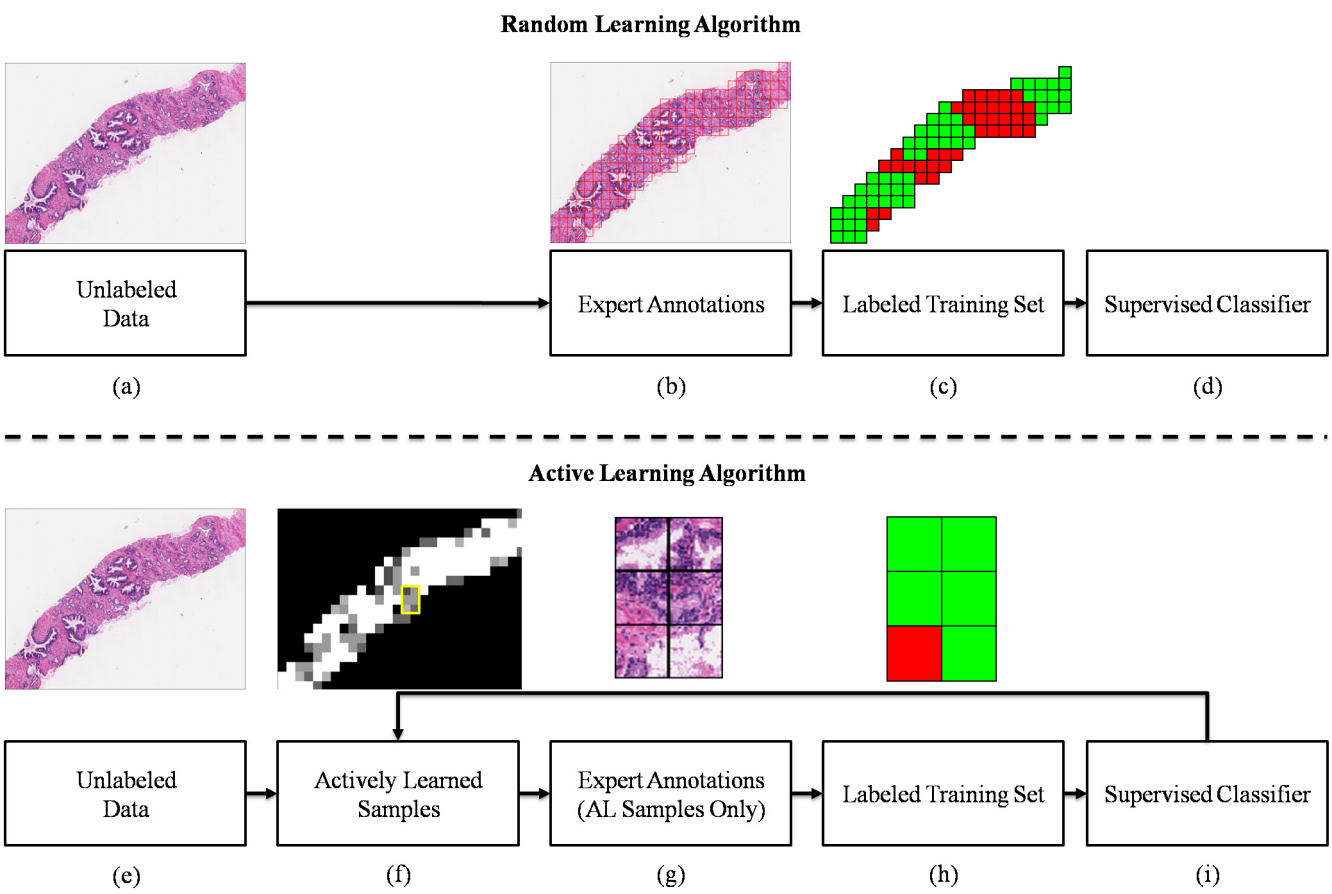
**Random Learning vs Active Learning Flowchart**. Comparison of Random Learning (RL, top row) and Active Learning (AL, bottom row) training processes. In RL, unlabeled data (a) is sent to an expert (b), who assigns a label to each sample in the image (c): red regions indicate cancer, and green indicates non-cancer. These labeled samples are used to train a supervised classifier (d). In AL, unlabeled samples (e) are analyzed to find informative samples (f), and only informative samples (g) are annotated for training (h). The supervised classifier (i) can be re-trained and used to identify new samples that may be informative. In the AL setup, only new samples that will improve classification accuracy are added.

The use of computers in histopathology analysis, known as digital pathology, is an increasingly common practice that promises to facilitate the detection, diagnosis, and treatment of disease [2]. Supervised classifiers have been applied in this context for a number of problems such as cancer detection and grading [3-8]. If the objective of the classifier is to distinguish normal from cancerous regions of tissue, exemplars corresponding to each class need to be manually labeled by a domain expert (typically a pathologist). Figure 1 shows an image from such an annotation task, where a prostate tissue sample stained with hematoxylin and eosin (H&E) has been digitized at 40× optical magnification using a whole-slide scanner. In this case, the goal of the supervised classifier is to identify regions of carcinoma of the prostate (CaP, the target class). The black contour in Figure 1 indicates the target class and was placed manually by an expert pathologist. We have previously shown [3] that a supervised classifier can accurately distinguish between CaP and non-CaP, but the annotation process required to build a large training set is laborious, time consuming, and expensive. The digitized images can be over 2 gigabytes (several million pixels) in size, making it difficult to quickly identify cancerous regions within the digital slide. In addition, CaP often appears within and around non-CaP areas, and the boundary between these regions is not always clear (even to a trained expert). These factors increase the time, effort, and overall cost associated with training a supervised classifier in the context of digital pathology. To reduce the cost and effort involved in training these classifiers, it is important to utilize an intelligent labeling strategy. In traditional supervised classification, samples are chosen from an unlabeled pool, annotated, and used to train a classification algorithm. This is known as random learning (RL), illustrated by the flowchart in Figure 2 (top row). In RL, no prior knowledge about the nature of the unlabeled samples is used, and it is possible that many non-informative samples (samples that will not have a positive impact on classifier performance) will be annotated; clearly a wasted effort. To improve training efficiency, a strategy known as active learning (AL) was developed to select only "informative" exemplars for annotation [9,10].

Informative samples are those which, if annotated and added to the training set, would increase the accuracy of the resulting trained classifier. In this setup, illustrated in Figure 2 (bottom row), the AL algorithm identifies informative samples (those which are difficult to classify) in an unlabeled dataset for annotation and addition to the growing training set. AL generates training sets that yield better classifier performance compared with training sets of the same size obtained via RL. The concept of "informative" samples in this context is related to the idea of margin-based classification in support vector machines (SVMs) [11], where labeled samples close to a decision boundary are used to classify unlabeled samples. In the AL context, informative samples are difficult-to-classify unlabeled data points that improve an existing training set.

Several AL algorithms have been proposed to determine whether an unlabeled sample is informative.

These methods measure the "informativeness" of a sample as the distance to a support-vector hyperplane [12,13], the disagreement among bagged weak classifiers [9,10], variation in feature distributions [14,15], and model-based predictions [16]. In a bioinformatics context, Lee, et al. [17] showed the benefits of using AL in building a naive Bayes classifier to identify disease states for several different datasets. Veeramachaneni, et al. [18] implemented an AL training approach to build a classifier identifying patient status from tissue microarray data. Previously [19], we investigated the performance of different AL algorithms in creating training sets for distinguishing diseased from non-diseases tissue samples.

Among the results of that study, we found that the particular AL algorithm chosen for learning had no significant effect on the performance of the supervised classifier.

Another major issue in supervised training involves the minority class problem, wherein the target class is under-represented in the dataset, relative to the non-target classes. A labeled training set comprises two sets of samples: representing training samples from the target (minority) class, and  being the samples from the non-target (majority) class. In the minority class problem, , where |·| indicates set cardinality. Several researchers [20-24] have shown that this training set will likely yield a classifier with lower accuracy and area under the receiver operating characteristic curve (AUC) compared with training sets where  or . Weiss and Provost [20] showed that for several datasets, varying the percentage of the minority class in the training set alters the accuracy and AUC of the resulting classifiers, and that the optimal class ratio was found to be significantly different from the "natural" ratio. Japkowicz and Stephen [21] found that the effect of the minority class problem depends on a number of factors, including the complexity of the target class and the size of the class disparity. Chawla, et al. [22] proposed mitigating the problem by over-sampling the minority class using synthetic samples; however, this method may simply introduce noise if the target class is too complex.

While some research has addressed the minority class problem in biomedical data [17,25], there has been little related work in the realm of digital pathology. Cosatto, et al. [26] applied a SVM AL method [12] in training a classifier for grading nuclear pleomorphism on breast tissue histology, while Begelman, et al. [27] employed an AL-trained SVM classifier in building a telepathology system for prostate tissue analysis. However, these studies did not account for the minority class problem in the training set, particularly relevant in the context of digital pathology, since the target class (cancer) is often observed far less often than the non-target class (non-cancer) and occupies only a small percentage of the overall tissue area. Ideally, an intelligent training strategy for this domain would combine AL while simultaneously addressing the minority class problem by maintaining a user-defined class ratio (class balancing). Zhu and Hovey [23] combined an entropy-based AL technique with over-and under-sampling to overcome the minority class problem for text classification, and found that over-sampling the minority class yielded the highest classifier performance. However, they did not investigate different class ratios and did not discuss the increased cost of the sampling techniques. Bloodgood and Vijay-Shanker [28] focused on an AL and classification method based on SVMs for unbalanced text and protein expression data; their approach involves estimating the class balance in the entire dataset, and then selecting samples to overcome this bias (as opposed to overcoming bias in the growing training set generated by AL).

While additional sampling can help to mitigate the minority class problem, this process requires more annotations compared to a training set with unbalanced classes. Because the cost of obtaining each annotation is high, it would be beneficial to be able to predict the number of annotations required to obtain a class-balanced training set of a pre-defined size. These predictions are critical for determining, *a priori*, the amount of resources (time, money, manpower) that will be employed in developing a supervised classifier. An analytical cost model will enable us to predict the cost involved in training the supervised classifier. Additionally, such a model will provide some insight into the relationship between (1) the size of a training set, (2) its class balance, and (3) the number of annotations required to achieve a predefined target accuracy.

### Contributions and Significance

In this work, we develop an AL-based classifier training strategy that also accounts for the minority class problem. This training strategy is referred to as "Class-Balanced Active Learning" (CBAL). We apply CBAL to the problem of building a supervised classifier to distinguish between CaP and non-CaP regions on images of prostate histopathology. For this particular problem, training samples are difficult and expensive to obtain, and the target class (CaP) is relatively sparse in relation to the non-target class; thus, we expect CBAL to yield large benefits in terms of training cost. Our mathematical model is used to predict the cost of building a training set of a pre-defined size and class ratio. This is, to the best of our knowledge, the first in-depth investigation and modeling of AL-based training for supervised classifiers that also specifically addresses the minority class problem in the context of digital pathology. However, CBAL training can be easily applied to other domains where obtaining annotated training samples is a time-consuming and difficult task, and where the target and non-target class ratios are not balanced. The rest of the paper is organized as follows. In Section 2 we describe the theory behind CBAL, followed by a description of the algorithms and model implementation in Section 3. In Section 4 we describe our experimental design, and in Section 5 we present the results and discussion. Concluding remarks are presented in Section 6.

## Methods

### Modeling the Annotation Cost of Class Balancing in Training

#### Notation and Symbols

A table containing commonly used notation and symbols is presented in Table 1. Our data comprises a set of square image regions *r *∈ *R *on digitized prostate images, represented by the red squares in Figure 2 (e). The regions *r *∈ *R *are divided into an unlabeled training pool, **S**^tr^, and an independent labeled testing pool, **S**^te^. Each sample has been identified as either belonging to the minority class *ω*_1 _(in this case the cancer class) or the majority (non-cancer) class, *ω*_2_. We denote membership of sample *r *∈ *R *in the minority class *ω*_1 _as *r ↪ ω*_1_, and these samples are "minority class samples." At iteration *t *∈ {0, 1, ⋯, *T *} of AL, the labeled training set is denoted as , where Φ denotes the training methodology and *T *is the maximum number of iterations. At each iteration *t*, a set of *M *weak binary classifiers is trained by  and used to build a strong classifier, . The selectivity of the AL algorithm is parameterized by *τ *∈ {0, ⋯, 0.5}, the confidence margin. We denote by  and  the desired number of samples *r *∈ *R *in the final training set for which *r ↪ ω*_1 _and *r ↪ ω*_2_, respectively. The total number of samples annotated at any iteration *t *is denoted as *N_t_*.

**Table 1:** Notation and Symbols

Symbol	Description	Symbol	Description
*r *∈ *R*	Dataset of image patches	*t *∈ {0, ⋯, *T*}	Iteration of *ActiveLearn*

**S**^tr^, **S**^te^	Unlabeled training, testing pools	Φ	Training methodology

$S_{t}^{\text{E}},{\hat{S}}_{t}^{\text{E}}$	Eligible samples, annotated samples	$S_{t,\Phi }^{\text{tr}}$	Samples labeled via Φ at *t*

$T_{t}$	Fuzzy classifier using	*k*_1,*t*_, *k*_2,*t*_	Number of samples in from *ω*_1_, *ω*_2_

*M*	Number of votes used to generate $T_{t}$	*ω*_1_, *ω*_2_	Possible classes of *r*

*τ*	Confidence margin	*r ↪ ω*_1_	Membership of *r *in class *ω*_1_

*θ *	Classifier-dependent threshold for $T_{t}$	$\hat{k_{1}},\hat{k_{2}}$	Number of samples in from *ω*_1_, *ω*_2_

*p_t_*(*r ↪ ω*_1_)	Probability of observing *r ↪ ω*_1_	*N_t_*	Samples added to training set at *t*

*P*_Δ_	Model confidence	${\hat{P}}_{t}$	Probability of observing samples

$A_{t}$	Accuracy of trained classifier at *t*		Total training cost after *T *iterations

List of the commonly used notation and symbols.

#### Theory of CBAL

In this subsection, we describe the theoretical foundation of the CBAL approach. Our goal in this section is to precisely define an "informative sample," identify the likelihood of observing a sample of a target class, and predict the number of samples that must be annotated before a specified number of target samples is observed and annotated. Our aim is to be able to predict *a priori *the cost of the system in terms of actively-learned annotations, which in turn represent an expenditure of resources.

**Definition 1**. *The set of informative samples (eligible for annotation)*, *, at any iteration t is given by the set of samples r *∈ *R for which *.

The value of  denotes the classification confidence, where  indicates strong confidence that *r ↪ ω*_1_, and  indicates confidence that *r ↪ ω*_2_. The number of samples  for which *r ↪ ω*_1 _and *r ↪ ω*_2 _are denoted *k*_1,*t *_and *k*_2,*t*_, respectively. The likelihood of randomly selecting a sample *r ↪ ω*_1 _from  is . The number annotated in class *ω*_2 _is .

**Proposition 1**. *Given the probability p_t_*(*r ↪ ω*_1_) *of observing a sample r ↪ ω*_1 _*at any iteration t, the probability **of observing **samples from class ω*_1 _*after annotating N_t _samples is:*

(1) $${\hat{P}}_{t}=\left(\begin{array}{c} N_{t}+\hat{k_{1}}-1\\ N_{t} \end{array}\right){\left[p_{t}\left(r↪\omega_{1}\right)\right]}^{N_{t}}{\left[\text{1}\;-p_{t}\left(r↪\omega_{1}\right)\right]}^{\hat{k_{1}}}$$

**Proof **Revealing the label of a sample  is an independent event resulting in either observation of class *ω*_1 _or *ω*_2_. The probability of success (i.e. observing a minority class sample) is *p_t_*(*r ↪ ω*_1_), and the probability of failure is *p_t_*(*r ↪ ω*_2_) = 1 - *p_t_*(*r ↪ ω*_1_) in the two class case. We assume that  is large, so *p_t_*(*r ↪ ω*_1_) is fixed. The annotations continue until  successes are achieved. Because of these properties, the number of annotations *N_t _*is therefore a negative binomial random variable, and the probability of observing  samples from class *ω*_1 _in *N_t _*annotations is given by the negative binomial distribution.

The consequence of Proposition 1 is that as *N_t _*(i.e. the training cost in annotations) increases,  also increases, indicating a greater likelihood of observing  samples *r ↪ ω*_1_. We denote as *P*_Δ _the target probability for the model to represent the degree of certainty that, within *N_t _*annotations, we have achieved our  samples *r *∈ *R *for which *r ↪ ω*_1_.

**Proposition 2**. *Given a target probability P*_Δ_*, the number of annotations required before **minority class samples are observed in ***S***^E ^is:*

(2) $$N_{t}=\underset{{\hat{k}}_{1}\leq x\leq |S^{tr}|}{\text{argmin}}\left[P_{\Delta }-\left(\begin{array}{c} x+{\hat{k}}_{1}-1\\ x \end{array}\right){\left[p_{t}(r\;≃\;\omega_{1})\right]}^{x}{\left[1\;-p_{t}(r\;≃\;\omega_{1})\right]}^{{\hat{k}}_{1}}\right].$$

**Proof **We wish to find the value of *N_t _*that causes Equation 1 to match our target probability, *P*_Δ_. When that happens,  and . Using a minimization strategy, we obtain the value of *N_t_*.

Proposition 2 gives us an analytical formulation for *N_t_*. Note that Equation 3 returns the smallest *N_t _*that matches the *P*_Δ_. The possible values of *N_t _*range from , in which case exactly  annotations are required, to *N_t _*= *|***S**^tr^*|*, in which case the entire dataset is annotated before obtaining > samples. Note that we are assuming that there are at least  samples in the unlabeled training set from which we are sampling.

### Algorithms and Implementation

#### AL Algorithm for Selecting Informative Samples

The CBAL training strategy consists of two algorithms that work in tandem: *ActiveTrainingStrategy*, for selecting informative samples, and *MinClassQuery*, for maintaining class balance. Algorithm *ActiveTrainingStrategy*, detailed below, requires a pool of unlabeled samples, **S**^tr^, from which samples will

**Algorithm ***ActiveTrainingStrategy*

**Input: S**^tr^, *T*

**Output: **, 

begin

0. initialization: create bootstrap training set , set *t *= 0

1. *while t < T do*

2.    Create classifier  from training set ;

3.    Find eligible sample set  where ;

4.    Annotate *K *eligible samples via *MinClassQuery() *to obtain ;

5.    Remove  from **S**^tr ^and add to ;

6.    *t *= *t *+ 1;

7. *endwhile*

8. *return *, ;

end

be drawn for annotation, as well as a parameter for maximum iterations *T*. This parameter can be chosen according to the available training budget or through a pre-defined stopping criterion. The output of the algorithm will be a fully annotated training set  as well as the classifier trained using training set . The identification of the informative samples occurs in Step 3, wherein a fuzzy classifier  is generated from a set of *M *weak binary decision trees [29] that are combined via bagging [30]. Informative samples are those samples for which half of the *M *weak binary decision trees disagree; that is, samples for which . This approach is similar to the Query-by-Committee (QBC) AL algorithm [9,10]. While there are several alternative algorithms available to perform AL-based training [12,14-16], we chose the QBC algorithm in this work due to its intuitive description of sample informativeness and its straightforward implementation. It is important to note that poor performance of  does not degrade the ability of the algorithm to identify informative samples. We expect that at low *t*, the performance of  will be low due to the lack of sufficient training, and much of the dataset will be identified as informative.

However, even if  identifies the majority of unlabeled samples as informative, it is still more efficient than RL. In the worst-case scenario, where all unlabeled samples are considered informative, then we are forced to choose training samples at random - which is equivalent to traditional supervised training.

#### Obtaining Annotations While Maintaining Class Balance

Algorithm *MinClassQuery *is used by *ActiveTrainingStrategy *to select samples from the set of eligible samples, , according to a class ratio specified by  and . Recall that , and so *K >*0. We expect that there will be many more samples from *ω*_2 _(the majority class) than from *ω*_1_. Because these

**Algorithm ***MinClassQuery*

**Input: **, *K *> 0, , 

Output: 

begin

0. initialization: , , 

1. *while **do*

2.    Find class *ω_i _*of a random sample , *i *∈ {1, 2};

3.    *if *

4.       Remove *r *from  and add to ****;

5.       ;

6.    *else*

7.       Remove *r *from ;

8.    *endif*

9. *endwhile*

10. *return *;

end

samples are being annotated, they are removed from the unlabeled eligible sample pool  in Step 7; however, since the resources have been expended to annotate them, they can be saved for future iterations.

#### Updating Cost Model and Stopping Criterion Formulation

At each iteration, we can calculate *N_t _*using Equation 1. We can estimate *p*_0_(*r ↪ ω*_1_) based on the size of the target class observed empirically from the initial training set (*<*10%); for *t >*0, we update the probability of observing a minority class sample using the following equation:

(3) $$p_{t+1}\left(r↪\omega_{1}\right)=\frac{k_{1,t}-\hat{k_{1}}}{k_{1,t}+k_{2,t}-N_{t}},$$

and *N_t+1 _*is re-calculated via the minimization of Equation 2. If {*r *∈ **S**^tr^*|r ↪ ω*_1_} = ⌀, then  and thus *p_t_*_+1_(*r ↪ ω*_1_) = 0. If there are no remaining samples in **S**^tr^, then *k*_1,*t *_+ *k*_2,*t *_= *N_t _*and *p_t+1_*(*r ↪ ω*_1_) is undefined. Essentially we must assume that (1) there are at least some samples *r *∈ **S**^tr ^for which *r ↪ ω*_1_, and (2) **S**^tr ^≠ ⌀. The cost of the entire training is calculated by summing *N_t _*for all *t*:

(4) $$ℒ=\overset{T}{\underset{t=1}{\sum }}N_{t}.$$

*ActiveTrainingStrategy *repeats until one of two conditions is met: (1) **S**^tr ^is empty, or (2) the maximum number of iterations *T *is reached. A stopping criterion can be trained off-line to determine the value of *T *as the smallest *t *that satisfies:

(5) $$|A_{t}-A_{t-1}|\leq \delta ,$$

where *δ *is a similarity threshold and  is the accuracy of classifier  (as evaluated on a holdout training set). Thus, when additional training samples no longer increase the resulting classifier's accuracy, the training can cease. An assumption in using this stopping criterion is that adding samples to the training set will not *decrease *classifier accuracy, and that accuracy will rise asymptotically. The total number of iterations *T *corresponds to the size of the final training set and can be specified manually or found using a stopping criterion discussed below. Classifiers that require a large training set will require a large value for *T*, increasing cost.

#### Selection of Free Parameters

Our methodology contains a few free parameters that must be selected by the user. The training algorithm employs three parameters: the similarity threshold *δ *(Equation 5); the confidence margin *τ*; and the number of samples from each class to add per iteration,  and . The choice of *δ *will determine the maximum number of iterations, *T*, the algorithm is allowed to run. A small value of *δ *will require a larger final training set (i.e. a larger *T*) before the algorithm satisfies the stopping criterion. Additionally, if Eq. 5 is never satisfied, then all available training samples will eventually be annotated (**S**^tr ^will be exhausted).

The confidence margin *τ *defines the range of values of  for which sample *r *is considered informative (difficult-to-classify). Smaller values of *τ *define a smaller area on the interval [0, 1], requiring more uncertainty for a region to be selected. *τ *= 0.0 indicates that only samples for which  (i.e. perfect classifier disagreement) are informative, while *τ *= 0.5 indicates that all samples are informative (equivalent to random learning). The number of samples to add from each class during an iteration of learning,  and , determines how many annotations occur before a new round of learning starts.

Consider the following two cases:

1. : in this case, 20 samples (10 from each class) are annotated per iteration.

2. : in this case, 2 samples (1 per class) are annotated per iteration.

In both cases, the learning algorithm for selecting informative samples is only updated after each iteration.

In the first case, 20 samples are added to  before new learning occurs, while in the second case, the learning algorithm is updated after each additional sample is annotated. Thus, in case 2, we are sure that each additional sample is chosen using the maximum amount of available information, while in case 1, several samples are added before the learning algorithm is updated. Although the second case requires ten iterations before it has the same training set size as the first case, each additional annotation is chosen based on an updated AL model, ensuring that all 20 samples are informative.

## Experimental Design

### Data Description

We apply the CBAL training methodology to the problem of prostate cancer detection from biopsy samples. Glass slides containing prostate biopsy samples are digitized at 40× magnification (0.25 *μ*m per pixel resolution). The original images are reduced in size using a pyramidal decomposition scheme [31] to 6.25% of their original size (4.0 *μ*m per pixel resolution), matching the resolution of the images used in [3]. Each image is divided into sets of square regions, *r *∈ *R *such that each region constitutes a 30-by-30 pixel square area (120-by-120 *μ*m area). These image regions constitute the dataset used for training and testing. Ground truth annotation is performed manually by an expert pathologist, who places a contour on tissue regions on the original 40× magnification image. Pathologists annotated both cancer and non-cancer regions of tissue, and only annotated regions were included in the dataset. A total of 100 biopsy images were analyzed from 58 patients, yielding over 12,000 annotated image regions. All of the 58 patients exhibited prostate cancer, although cancer was not present in all 100 images. The square regions were assumed to be independently drawn from the images.

### Feature Extraction

**Figure 3:**
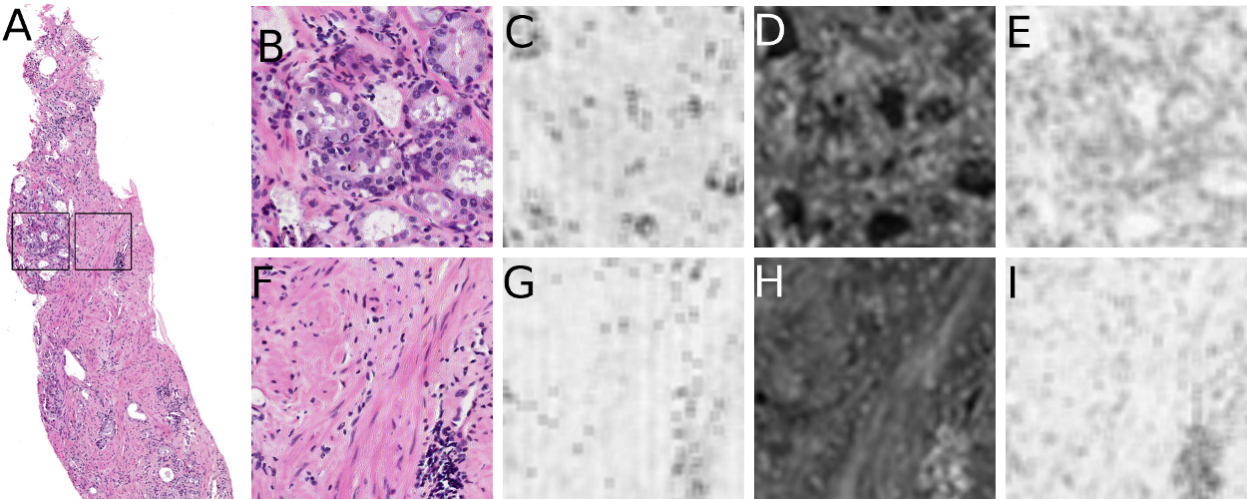
**Examples of Feature Types**. Examples of the feature types extracted on two ROIs from a biopsy sample (a), identified by black squares. Shown are (b), (f) the original tissue image, (c), (g) a greylevel texture image (standard deviation value), (d), (h) a Haralick texture image (entropy of the co-adjacency matrix), and (e), (i) a Gabor filter feature image. The top row (b)-(e) indicates a cancerous region, while the bottom row (f)-(i) is a benign region.

In [3], we built a classifier for discriminating between cancer and non-cancer on a pixel level. We extracted several hundred texture features, comprising three different classes of texture descriptors: Grey-level statistics of image intensities, Haralick texture features based on the co-adjacency of image intensities, and Gabor filter features based on a filter bank utilizing phase and scale parameters. Examples of these feature types are given in Figure 3. We employed the Adaptive Boosting (AdaBoost) algorithm [32], which is a method of assigning a weight to each feature based on its discriminating power. Features with a higher weight are better able to capture the differences between classes; a subset of highly informative features can be selected as those with weights above 0.05. In the current study, we employed those 14 features under the assumption that the features useful for pixel-wise classification would be similarly useful in patch-wise classification of cancer. The feature values were calculated in a pixel-wise fashion for each 30-by-30 region, and each region *r *was then represented by the average value of the feature calculated over all pixels.

#### First-order Statistical Features

First-order features are statistics calculated directly from the pixel values in the image. These include the mean, median, and standard deviation of the pixels within a window size, as well as Sobel filters and directional gradients. Of these features, two were included in the subset: the standard deviation and the range of pixel intensities.

#### Second-order Co-occurrence Features

Co-occurrence image features are based on the adjacency of pixel values in an image. An adjacency matrix is created where the value of the *i*th row and the *j*th column equals the number of times pixel values *i *and *j *appear within a fixed distance of one another. A total of thirteen Haralick texture features [33] are calculated from this co-adjacency matrix, of which 5 were found to be highly discriminating: information measure, correlation, energy, contrast variance, and entropy.

#### Steerable Filter Features

To quantify spatial and directional textures in the image, we utilize a steerable Gabor filter bank [34]. The Gabor filter is parameterized by frequency and orientation (phase) components; when convolved with an image, the filter provides a high response for textures that match these components. We compute a total of 40 filter banks, of which 7 were found to be informative, from a variety of frequency and orientation values.

### Evaluation of Training Set Performance via Probabilistic Boosting Trees

Evaluation of  is done by testing the trained classifier's accuracy. To avoid biasing the results, we wish to use a different classifier than  for evaluation; a probabilistic boosting tree (PBT) [35], denoted , is employed. The PBT combines AdaBoost [32] and decision trees [29] and recursively generates a decision tree where each node is boosted with *M *weak classifiers. The classifier output, , is the probability that sample *r *belongs to the target class. The PBT is used to classify an independent testing set **S**^te ^(where **S**^te ^∩ **S**^tr ^= ⌀) via area under the receiver operating characteristic curve (AUC) and classifier accuracy. The hard classification for *r *∈ **S**^te ^is denoted as:

(6) $${\tilde{T}}_{t}\left(r\right)=\left\{\begin{array}{cc} 1 & \text{if }{\tilde{T}}_{t}\left(r\right)>\theta \\ 0 & \text{otherwise}, \end{array}\right)$$

where *θ *is a classifier-dependent threshold. For region *r*, the ground truth label is denoted as , where a value of 1 indicates class *ω*_1 _and 0 indicates class *ω*_2_. The resulting accuracy at iteration *t *is denoted as:

(7) $$A_{t}=\frac{1}{\left|R\right|}\underset{r}{\sum }\left\{\begin{array}{cc} 1 & \text{if }G\left(r\right)={\tilde{T}}_{t}\left(r\right)\\ 0 & \text{otherwise}. \end{array}\right)$$

We generate receiver operating characteristic (ROC) curves by calculating the classifier's sensitivity and specificity at various decision thresholds *θ *∈ {0, ..., 1}. Each value of *θ *yields a single point on the ROC curve, and the area under the curve (AUC) measures the discrimination between cancer and non-cancer regions. The accuracy can then be calculated by setting *θ *to the operating point of the ROC curve. Again, it should be noted that it is possible to evaluate the performance of the training set using any supervised classifier in place of PBT. A previous study [36] used both decision trees [29] and SVMs [11] as supervised evaluation algorithms in an AL training experiment, and found that the trend in performance for both algorithms was similar. In this study we implemented PBTs because the algorithm was different from , which avoids biasing results; however, alternative evaluation algorithms could certainly be employed.

Although the classifier performance values may change, the goal of these experiments is to show that the performance of an actively-learned, class-balanced training set is better than a randomly generated unbalanced set.

### List of Experiments

We perform three sets of experiments to analyze different facets of the active learning training methodology.

**Experiment 1: Comparison of CBAL Performance with Alternate Training Strategies **We compare the performance of CBAL with four alternative training strategies to show that CBAL training will yield a classifier with greater performance versus a training set of the same size trained using an alternative method.

• Unbalanced Active Learning (UBAL): The class ratio is not controlled; eligible samples  determined via AL are randomly annotated and added to .

• Class Balanced Random Learning (CBRL): All unlabeled samples in **S**^tr ^are eligible for annotation, while holding class balance constant as described in *MinClassQuery*.

• Unbalanced Random Learning (UBRL): All unlabeled samples are queried randomly. This is the classic training scenario, wherein neither class ratio nor informative samples are explicitly controlled.

• Full Training (Full): All available training samples are used. This represents the performance when the entire training set is annotated and available (an ideal scenario).

In random learning (RL), all samples in the unlabeled pool **S**^tr ^are "eligible" for annotation; that is, **S**^E ^= **S**^tr^. In unbalanced class experiments, the *MinClassQuery *algorithm is replaced by simply annotating *K *random samples (ignoring class) and adding them to . The full training strategy represents the scenario when all possible training data is used.

The classifier is tested against an independent testing pool, **S**^te^, which (along with the training set) is selected at random from the dataset at the start of each trial. In these experiments, *T *= 40, the confidence margin was *τ *= 0.25, and the number of samples added at each iteration was *K *= 2. In the balanced experiments, . A total of 12,588 image regions were used in the overall dataset, drawn from the 100 images in the dataset; 1,346 regions were randomly selected for **S**^te^, and 11,242 for **S**^tr ^in each of 10 trials. The regions are assumed to be independent samples of the overall image space due to the heterogeneity of the tissue and appearance of disease. Because the goal of classification is to distinguish between cancer and non-cancer regions of tissue rather than individual patients, the training and testing was drawn randomly from the overall pool of available regions. The true ratio of non-cancer to cancer regions in **S**tr was approximately 25:1 (4% belonged to the cancer class). A total of 10 trials were performed, with random selection of **S**^tr ^and **S**^te ^at the beginning of each trial.

**Experiment 2: Effect of Training Set Class Ratio on Accuracy of Resulting Classifier **To explore the effect of training set class ratio on the performance of the resulting classifier, the CBAL methodology was used, setting *K *= 10 and varying  and  such that the percentages of the training set consisting of minority samples vary from 20%  to 80% . Each set of parameters was used to build a training set, which in turn was used to build a classifier that was evaluated on the same independent testing set **S**^te^.

**Experiment 3: Comparison of Cost Model Predictions with Empirical Observations **At each step of the AL algorithm, we estimate *N_t _*for obtaining balanced classes as described in Section 2. The goal of this experiment was to empirically evaluate whether our mathematical model could accurately predict the cost of obtaining balanced classes at each iteration, and could thus be used to predict the cost of classifier training for any problem domain. For these calculations, we set the initial class probability *p*_0_(*ω*_1_) = 0.04, based on the observations of the labeled data used at the beginning of the AL process. Additionally, we set the desired sample numbers to correspond with the different class ratios listed in Experiment 2, from 20% minority class samples  to 80% . The aim of this experiment was to investigate the relationship between the cost of a specific class ratio and the performance of >.

## Results and Discussion

### Experiment 1: Comparison of CBAL performance with Alternate Training Strategies

**Figure 4:**
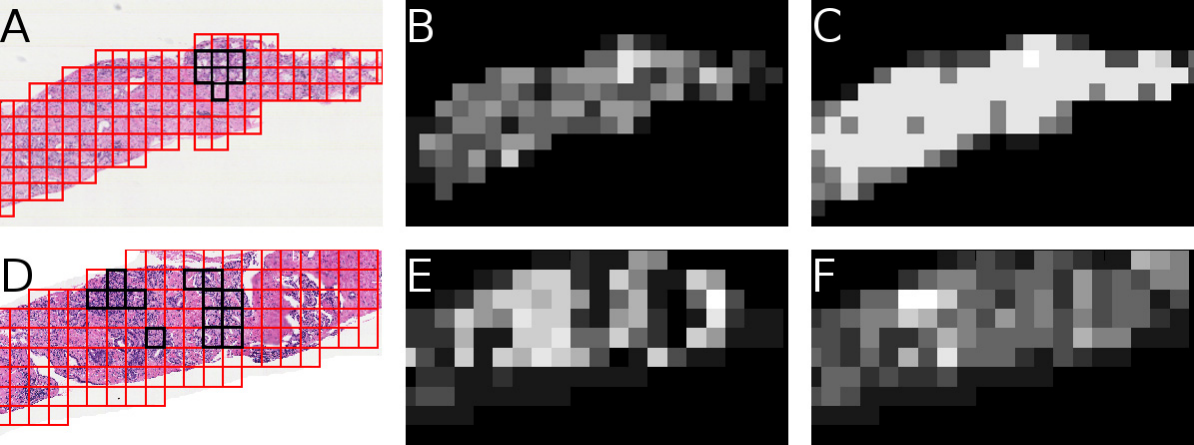
**Qualitative Results of the Probabilistic Boosting Tree Classifier**. Qualitative results of the final PBT classifier . Shown in (a), (d) are the segmented cancer region, (b), (e) show the probability scene obtained through the CBAL classifier, and (c), (f) show the probability scene obtained via CBRL. The intensity of a region is determined by .

Examples of confidence or likelihood scenes generated by  are shown in Figure 4, obtained at iteration *T *= 40 (since *K *= 2, these images represent the classifier's performance using 80 total samples). Figures 4(a) and 4(d) show images with benign regions marked in red boundaries and cancerous regions in black. Figures 4(b) and 4(e) show the confidence scenes obtained via the CBAL training strategy, and (c) and (f) are obtained via CBRL training. High intensity regions represent high classifier confidence that *r ↪ ω*_1_, while dark regions indicate confidence that *r ↪ ω*_2_. In both cases, the CBRL training fails to properly find the cancer regions, either returning large numbers of false positives (Figure 4(c)) or failing to fully identify the cancer area (Figure 4(f)). This difference (high false positives in one case, high false negatives in another) is most likely due to the inability of random learning to accurately define the classes, given the small training set size. Thus, given the constraints on training set size, a CBAL-trained classifier can out-perform a randomly-trained classifier.

**Figure 5:**
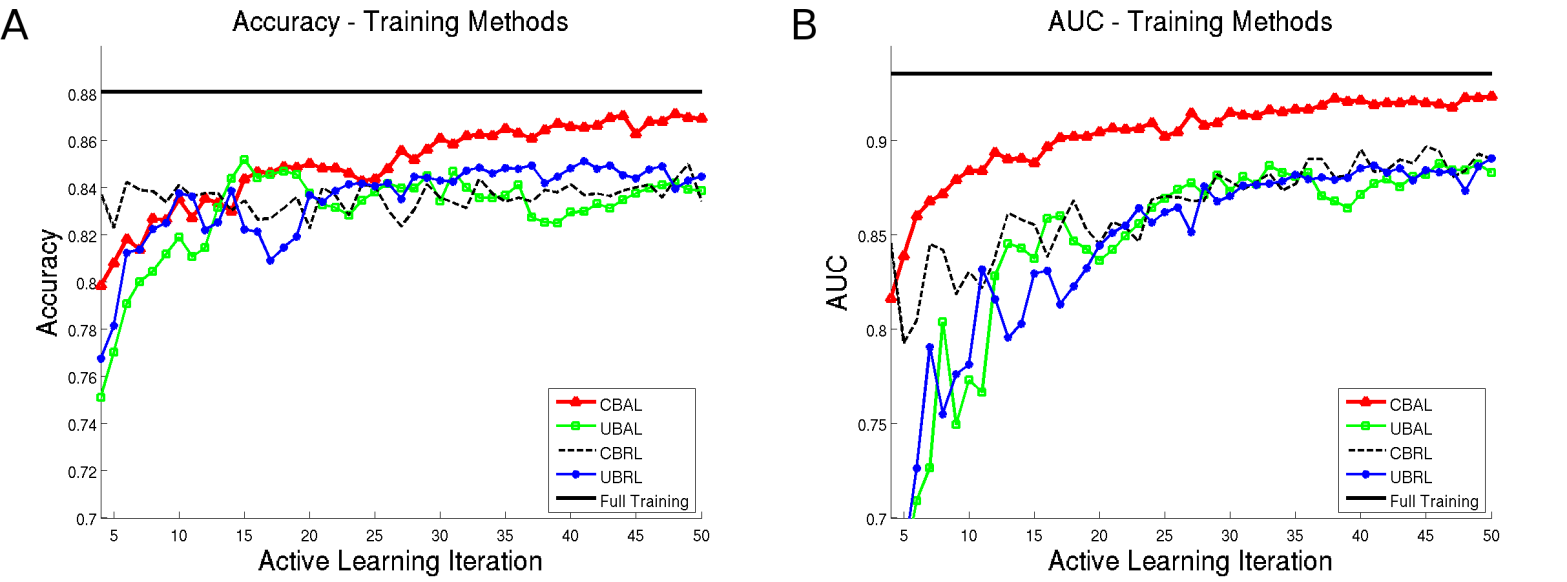
**Quantitative Results of the Probabilistic Boosting Tree Classifier**. Quantitative results of the classifier, , for *t *∈ {1, 2, ⋯, *T*}. Shown are (a) accuracy and (b) AUC values for the PBT classifier, evaluated at each iteration.

Quantitative classification results are plotted in Figure 5 as accuracy (Figure 5(a)) and area under the ROC curve (Figure 5(b)) as a function of the number of training samples in the set  for 1 ≤ *t *≤ 40. In each plot, the "full" training set corresponds to the straight black line, CBAL is the red triangle line, CBRL is a black dashed line, UBAL is a green squared line, and UBRL is a blue circled line. Note that the "full" line indicates the maximum achievable classifier accuracy for a given training set; thus, the closer a training set gets to the straight black line, the closer it is to optimal performance.

The AUC values for CBAL approach the full training with 60 samples (*t *= 30) while CBRL, UBRL, and UBAL have approximately 0.05 lower AUC at those sample sizes. Accuracy for CBAL remains similar to other methods until *t *= 30, at which point CBAL out-performs other methods by approximately 3%. CBRL, UBRL, and UBAL do not perform as well as CBAL for the majority of our experiments, requiring a larger number of samples to match the accuracy and AUC of CBAL.

### Experiment 2: Effect of Training Set Class Ratio on Accuracy of Resulting Classifier

**Figure 6:**
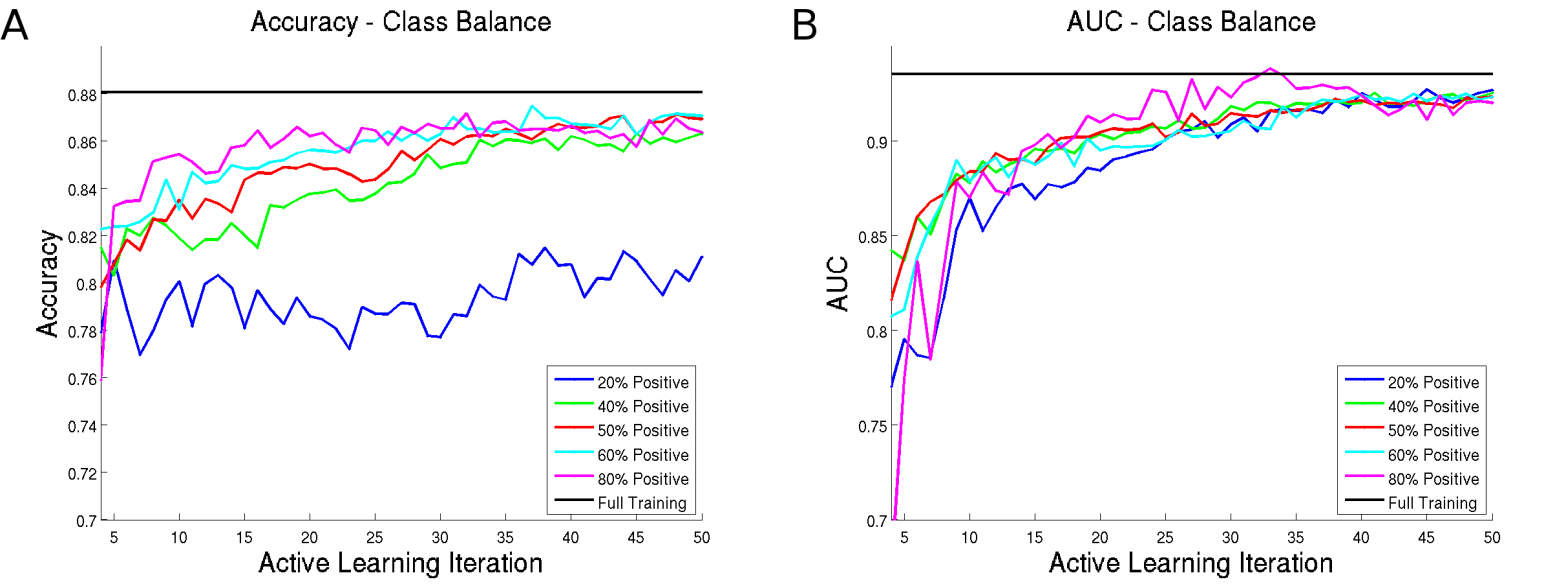
**Performance of the PBT Classifier Trained on Different Target Class Ratios**. Performance of the PBT classifier trained using training sets with different percentages of samples for which *r ↪ ω*_1_. Shown are the (a) accuracy and (b) AUC values for the trained classifier at each iteration, using *p*_0_(*r ↪ ω*_1_) = 0.04.

Figure 6 shows the effects of varying training class ratios on the resulting classifier's performance for the prostate cancer detection problem. Shown is the performance of the PBT classifier at each iteration of the AL algorithm using 20% minority samples (blue line), 40% (green line), 50% (red line), 60% (cyan line), and 80% (magenta line), for both accuracy (Figure 6(a)) and AUC (Figure 6(b)). The AUC curves are similar for all class ratios, although the training set that uses 80% minority class samples tends to perform slightly better. Thus, by over-representing the minority class, we achieve greater performance in terms of accuracy. Noted that while changing the class ratio had different effects on accuracy and AUC a similar trend was reported by Weiss and Provost [20], who found that altering the class ratio of a training set for a classifier affected AUC and accuracy differently (although there was no specific trend across multiple datasets).

### Experiment 3: Comparison of Cost Model Predictions with Empirical Observations

**Figure 7:**
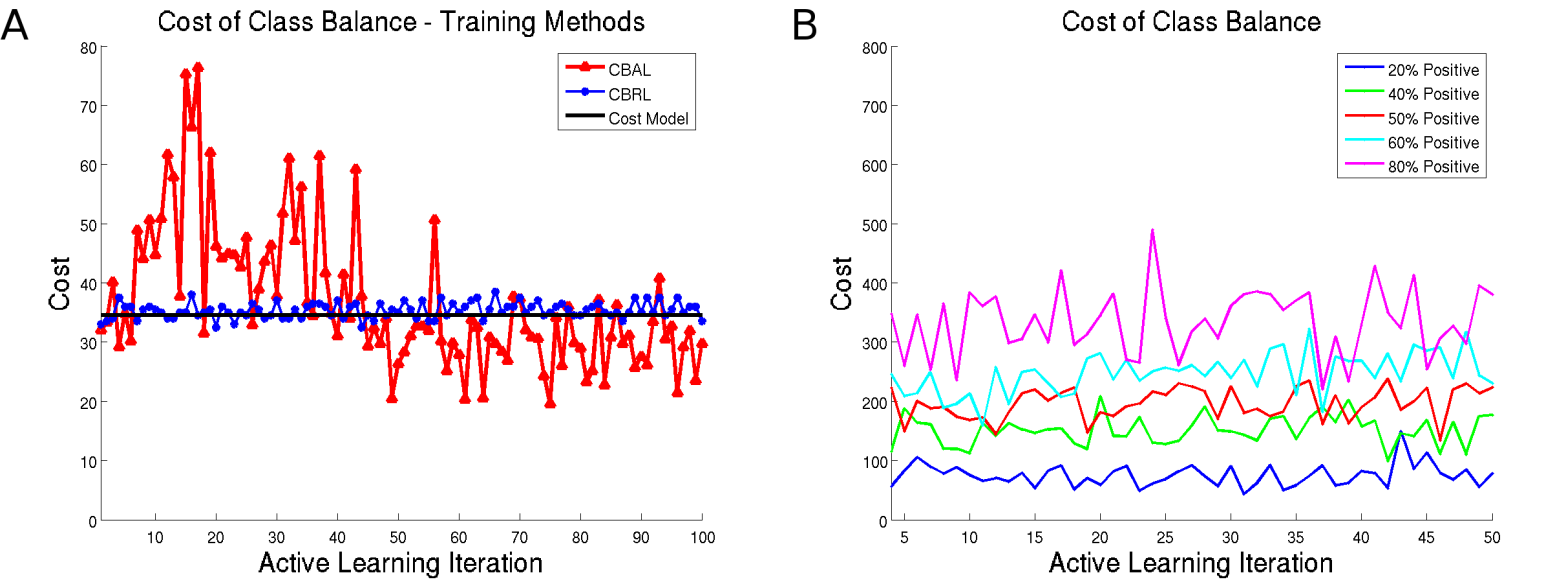
**Cost of Class Balance**. (a) Plot of annotations *N_t _*required for class balance as a function of *t*; shown are CBAL (blue line), CBRL (red dashed line), and the predicted *N_t _*from Equation 1 (black line). (b) The cost of obtaining a specific class ratio as iterations increase. If a high percentage of minority class samples is desired, the cost increases.

Figure 7(a) shows the results of cost modeling simulations. The predicted cost, found by solving for *N_t _*in Equation 1, is plotted as a function of *t *(solid black line) with *p*_0_(*r ↪ ω*_1_) = 0.04 along with the empirically observed costs of CBRL (blue dotted line) and CBAL (red triangle line) with . At each *t*, the plots show how many annotations were required before class balancing was achieved. We can see that the simulation predicts the number of annotations required to achieve class balance at each iteration within approximately 10-20 annotations. Additionally, we see that the empirically observed costs are greatly varied, particularly for *t <*50; this is due to the fact that the number of annotations required to achieve class balance depends greatly on (1) the current training set, (2) the remaining samples in the unlabeled pool, and (3) the order in which eligible samples are chosen for annotation.

While it may seem from Figure 6 that the strategy yielding best performance would be to over-sample the minority class as much as possible, we also plotted the empirical cost values *N_t _*for each of the class ratios from Experiment 2 in Figure 7(b). We find that as the percentage of the minority class increases, the cost associated with each iteration of the AL algorithm also increases. This is due to the fact that as the minority class is over-sampled, more annotations are required to find additional minority samples. While there is some increase in accuracy by over-sampling the dataset, the annotation cost increases by an order of magnitude. Thus, the optimal strategy will need to balance the increase in accuracy with the constraints of the overall annotation budget.

## Conclusions

In this work we present a strategy for training a supervised classifier when the costs of training are high, and where the minority class problem exists. Our strategy, Class-Balanced Active Learning (CBAL), has the following characteristics: (1) Active Learning (AL) is used to select informative samples for annotation, thus ensuring that each annotation is highly likely to improve classifier performance. (2) Class ratios are specifically addressed in this training strategy to prevent the training set from being biased toward the majority class. (3) A mathematical model is used to predict the number of annotations required before the specified class balance is reached. We applied these techniques to the task of quantitatively analyzing digital prostate tissue samples for presence of cancer, where the CBAL training method yielded a classifier with accuracy and AUC values similar to those obtained with the full training set using fewer samples than the unbalanced AL, class-balanced random learning, or unbalanced random learning methods. Our mathematical cost model was able to predict the number of annotations required to build a class-balanced training set within 20 annotations, despite the large amount of variance in the empirically observed costs. This model is critical in determining, *a priori*, what the cost of training will be in terms of annotations, which in turn translates into the time and effort expended by the human expert in helping to build the supervised classifier. We found that by specifying class ratios for the training set that favor the minority class (i.e. over-sampling), the resulting classifier performance increased slightly; however, the cost model predicted a large increase in the cost of training, as a high percentage of minority class samples requires more annotations to build. Thus, an optimal training strategy must take into account the overall training budget and the desired accuracy.

Some of the specific findings in this work, such as the observation that over-representing the minority class yields a slightly higher classifier performance, may be specific to the dataset considered here. Additionally, the observation that the AL algorithm has a large amount of variance in the empirically-observed costs (particularly at the beginning of training) indicates that the eligible sample set is unpredictable with respect to class compositions. This behavior may not necessarily be duplicable with different datasets or AL strategies, both of which will yield eligible sample sets with different class compositions. Additionally, we do not claim that our choice of AL algorithm (QBC), our weak classification algorithm (bagged decision trees), or our evaluation classifier (PBT) will out-perform the available alternatives. However, by combining AL and class balancing, we have developed a general training strategy that should be applicable to most supervised classification problems where the dataset is expensive to obtain and which suffers from the minority class problem. These problems are particularly prevalent in medical image analysis and digital pathology, where the costs of classifier training are very high and an intelligent training strategy can help save great amounts of time and money. Training is an essential and difficult part of supervised classification, but the integration of AL and intelligent choice of class ratios, as well as the application of a general cost model, will help researchers to plan the training process more quickly and effectively. Future work will involve extensions of our framework to the multi-class case, where relationships between multiple classes with different distributions must be taken into account.

## Authors' contributions

SD processed the dataset, developed the training algorithm and cost model theory, ran the experiments, analyzed the results and wrote the manuscript. JM assisted with developing the training algorithm and cost model theory, as well as writing the manuscript. JT and MF provided the dataset, as well as annotations and medical insights into the data. AM directed the research and the development of the manuscript. All authors have read and approved the final manuscript.

## References

[B1] Van der Walt C Barnard E Data Characteristics that Determine Classifier Performance17th Annual Symposium of the Pattern Recognition Association of South Africa 2006 6 12

[B2] Gurcan M Boucheron L Can A Madabhushi A Rajpoot N Yener B Histopathological Image Analysis: A Review IEEE Reviews in Biomedical Engineering 2009 2 147 171 20671804 10.1109/RBME.2009.2034865 PMC2910932

[B3] Doyle S Feldman M Tomaszewski J Madabhushi A A Boosted Bayesian Multi-Resolution Classifier for Prostate Cancer Detection from Digitized Needle Biopsies IEEE Transactions on Biomedical Engineering (In Press, PMID 20570758) 2010 10.1109/TBME.2010.2053540 20570758

[B4] Madabhushi A Doyle S Lee G Basavanhally A Monaco J Masters S Feldman M Tomaszewski J Review: Integrated Diagnostics: A Conceptual Framework with Examples Clinical Chemistry and Laboratory Medicine 2010 989 998 10.1515/CCLM.2010.193 20491597

[B5] Doyle S Agner S Madabhushi A Feldman M Tomaszewski J Automated Grading of Breast Cancer Histopathology Using Spectral Clustering with Textural and Architectural Image Features ISBI 2008. 5th IEEE International Symposium 2008 496 499

[B6] Monaco J Tomaszewski J Feldman M Hagemann I Moradi M Mousavi P Boag A Davidson C Abolmaesumi P Madabhushi A High-throughput detection of prostate cancer in histological sections using probabilistic pairwise Markov models Medical Image Analysis 2010 14 4 617 629 10.1016/j.media.2010.04.007 20493759 PMC2916937

[B7] Fatakdawala H Xu J Basavanhally A Bhanot G Ganesan S Feldman M Tomaszewski J Madabhushi A Expectation Maximization driven Geodesic Active Contour with Overlap Resolution (EMaGACOR): Application to Lymphocyte Segmentation on Breast Cancer Histopathology Biomedical Engineering, IEEE Transactions on 2010 57 7 1676 1689 10.1109/TBME.2010.2041232 20172780

[B8] Basavanhally A Ganesan S Agner S Monaco J Feldman M Tomaszewski J Bhanot G Madabhushi A Computerized Image-Based Detection and Grading of Lymphocytic Infiltration in HER2+ Breast Cancer Histopathology Biomedical Engineering IEEE Transactions on 2010 57 3 642 653 10.1109/TBME.2009.2035305 19884074

[B9] Seung H Opper M Smopolinsky H Query by committee Proceedings of the 5th Annual ACM Workshop on Computational Learning Theory 1992 287 294

[B10] Freund Y Seung H Shamir E Tishby N Selective Sampling Using the Query by Committee Algorithm Machine Learning 1996 28 133 168

[B11] Cortes C Vapnik V Support-Vector Networks Machine Learning 1995 20 273 297

[B12] Tong S Koller D Active Learning for Structure in Bayesian Networks 2001

[B13] Li M Sethi IK Confidence-based active learning IEEE Transactions on Pattern Analysis and Machine Intelligence 2006 28 8 1251 61 [Journal Article United States] 16886861 10.1109/TPAMI.2006.156

[B14] Cohn D Atlas L Ladner R Improving generalization with active learning Machine Learning 1994 15 2 201 221 [10.1007/BF00993277]

[B15] Cohn D Ghahramani Z Jordan M Active Learning with Statistical Models Journal of Artificial Intelligence Research 1996 4 129 145

[B16] Schmidhuber J Storck J Hochreiter S Reinforcement Driven Information Acquisition in Non-Deterministic Environments Tech report, Fakultät für Informatik, Technische Universität München 1995 2 159 164

[B17] Lee M Rhee J Kim B Zhang B AESNB: Active Example Selection with Naive Bayes Classifier for Learning from Imbalanced Biomedical Data 2009 Ninth IEEE International Conference on Bioinformatics and Bioengineering 2009 15 21

[B18] Veeramachaneni S Demichelis F Olivetti E Avesani P Jorge A, Torgo L, Brazdil P, Camacho R, Gama J Active Sampling for Knowledge Discovery from Biomedical Data Knowledge Discovery in Databases: PKDD 2005, Volume 3721 of Lecture Notes in Computer Science 2005 Springer Berlin/Heidelberg 343 354

[B19] Doyle S Madabhushi A Consensus of Ambiguity: Theory and Application of Active Learning for Biomedical Image Analysis Pattern Recognition in Bioinformatics (PRIB) 2010

[B20] Weiss GM Provost F The Effect of Class Distribution on Classifier Learning: An Empirical Study Technical Report ML-TR-44 2001 http://citeseerx.ist.psu.edu/viewdoc/summary?doi=?doi=10.1.1.28.9570

[B21] Japkowicz N Stephen S The Class Imbalance Problem: A Systematic Study Intelligent Data Analysis 2002 6 429 449

[B22] Chawla N Bowyer K Hall L Kegelmeyer W SMOTE: Synthetic Minority Over-sampling Technique Journal of Artificial Intelligence Research 2002 16 321 357

[B23] Zhu J Hovy E Active Learning for Word Sense Disambiguation with Methods for Addressing the Class Imbalance Problem Proceedings of the 2007 Joint Conference on Empirical Methods in Natural Language Processing and Computational Natural Language Learning (EMNLP-CoNLL) 2007 Prague, Czech Republic: Association for Computational Linguistics 783 790 http://www.aclweb.org/anthology/D/D07/D07-1082

[B24] Batista G Carvalho A Monard M Cairo O, Sucar L, Cantu F Applying One-Sided Selection to Unbalanced Datasets MICAI 2000: Advances in Artificial Intelligence, Volume 1793 of Lecture Notes in Computer Science 2000 Springer Berlin/Heidelberg 315 325

[B25] Yang K Cai Z Li J Lin G A stable gene selection in microarray data analysis BMC Bioinformatics 2006 7 228 http://www.biomedcentral.com/1471-2105/7/228 10.1186/1471-2105-7-228 16643657 PMC1524991

[B26] Cosatto E Miller M Graf H Meyer J Grading nuclear pleomorphism on histological micrographs Pattern Recognition, ICPR 2008. 19th International Conference on 2008 2008 1 4

[B27] Begelman G Pechuk M Rivlin E A Microscopic Telepathology System for Multiresolution Computer-Aided Diagnosis Journal of Multimedia 2006 1 7 40 48

[B28] Bloodgood M Vijay-Shanker K Morristown, NJ Taking into account the differences between actively and passively acquired data: the case of active learning with support vector machines for imbalanced datasets NAACL '09: Proceedings of Human Language Technologies: The 2009 Annual Conference of the North American Chapter of the Association for Computational Linguistics, Companion Volume: Short Papers 2009 USA: Association for Computational Linguistics 137 14017662975

[B29] Quinlan J Quinlan J Decision trees and decision-making IEEE Trans Syst Man Cybern 1990 20 2 339 346 10.1109/21.52545

[B30] Breiman L Bagging Predictors Machine Learning 1996 24 2 123 140

[B31] Burt P Adelson E The Laplacian Pyramid as a Compact Image Code Journal of Communication 1983 31 4 532 540

[B32] Freund Y Schapire R Experiments with a New Boosting Algorithm Machine Learning: Proceedings of the Thirteenth International Conference 1996 148 156

[B33] Haralick R Shanmugan K Dinstein I Textural features for image classification IEEE Trans on Systems Man and Cybernetics 1973 SMC-3 610 621

[B34] Manjunath B Ma W Texture features for browsing and retrieval of image data Transactions on Pattern Analysis and Machine Intelligence 1996 18 (8):837 842

[B35] Tu Z Probabilistic boosting-tree: Learning discriminative models for classification, recognition, and clustering ICCV 2005 2 1589 1596

[B36] Doyle S Monaco J Feldman M Tomaszewski J Madabhushi A A Class Balanced Active Learning Scheme that Accounts for Minority Class Problems: Applications to Histopathology OPTIMHisE Workshop (MICCAI) 2009 19 30

